# An amphiphilic region in the cytoplasmic domain of KdpD is recognized by the signal recognition particle and targeted to the *Escherichia coli* membrane

**DOI:** 10.1111/j.1365-2958.2008.06246.x

**Published:** 2008-06

**Authors:** Katja S Maier, Stefanie Hubich, Helga Liebhart, Susanne Krauss, Andreas Kuhn, Sandra J Facey

**Affiliations:** Institute of Microbiology, University of Hohenheim70599 Stuttgart, Germany

## Abstract

The sensor protein KdpD of *Escherichia coli* is composed of a large N-terminal hydrophilic region (aa 1–400), four transmembrane regions (aa 401–498) and a large hydrophilic region (aa 499–894) at the C-terminus. KdpD requires the signal recognition particle (SRP) for its targeting to the membrane. Deletions within KdpD show that the first 50 residues are required for SRP-driven membrane insertion. A fusion protein of the green fluorescent protein (GFP) with KdpD is found localized at the membrane only when SRP is present. The membrane targeting of GFP was not observed when the first 50 KdpD residues were deleted. A truncated mutant of KdpD containing only the first 25 amino acids fused to GFP lost its ability to specifically interact with SRP, whereas a specific interaction between SRP and the first 48 amino acids of KdpD fused to GFP was confirmed by pull-down experiments. Conclusively, a small amphiphilic region of 27 residues within the amino-terminal domain of KdpD (aa 22–48) is recognized by SRP and targets the protein to the membrane. This shows that membrane proteins with a large N-terminal region in the cytoplasm can be membrane-targeted early on to allow co-translational membrane insertion of their distant transmembrane regions.

## Introduction

The KdpD protein of *Escherichia coli* is a membrane component of the KdpD/E sensory system involved in maintaining the intracellular osmolarity. In cases of low K^+^ concentrations in the medium, the expression of the high-affinity K^+^ pump KdpFABC is induced (Epstein and Davis, 1970). In this signalling cascade, the C-terminal domain of KdpD is phosphorylated most likely at His673 (Voelkner *et al.*, 1993). Subsequently, the phosphoryl group is transferred to Asp52 of the cytoplasmic response regulator KdpE, which in turn acts as a transcriptional activator for *kdpFABC* (Sugiura *et al.*, 1992; Nakashima *et al.*, 1993). Although the exact mechanism of the K^+^ sensing is still unknown, the C-terminal domain of KdpD is by itself functional (Rothenbücher *et al.*, 2006).

**Fig. 1:**
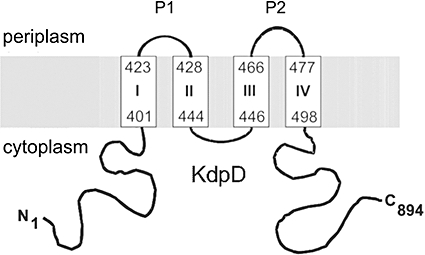
Membrane topology of KdpD. The KdpD protein consists of 894 amino acid residues organized as two hydrophilic domains that are separated by four closely spaced transmembrane regions. The periplasmic loops of KdpD (P1 + P2) contain 4 and 10 residues, respectively.

The biosynthesis of KdpD has been studied in detail. The KdpD protein consists of a large cytoplasmic N-terminal domain, four closely spaced transmembrane regions, and an extended cytoplasmic C-terminal domain (Fig. 1). The insertion of the transmembrane regions occurs independently of SecA, SecE and YidC, but involves the electrochemical membrane potential (Facey and Kuhn, 2003). As the translocated periplasmic region P1 between the first and second transmembranes is only four residues long, it was extended to 19 residues with an antigenic epitope. The mutant showed the same insertion characteristics for the translocation of the P1 region as the wild-type protein and was Sec- and YidC-independent. It was therefore concluded that short periplasmic loops can translocate without assistance of other known proteins (Facey and Kuhn, 2003).

In *E. coli,* there are multiple pathways for directing proteins to and across the inner membrane. The twin-arginine translocation system (Tat) targets a small group of cofactor-containing proteins across the cytoplasmic membrane via distinct signal peptides bearing a conserved RR motif (Alami *et al.*, 2003). The Tat pathway transports only folded proteins post-translationally through the Tat translocon. The majority of secreted *E. coli* proteins are synthesized as preproteins with a cleavable signal peptide at their N-terminus. These preproteins are targeted to the cytoplasmic membrane post-translationally by the molecular chaperone SecB via the cytosolic ATPase SecA which is associated with the SecYEG translocase (Driessen *et al.*, 2001). The core of the Sec translocon consists of the integral membrane proteins SecY, SecE and SecG, and the peripheral subunit SecA. The Sec translocase mediates the stepwise translocation of secretory proteins across the membrane. In contrast, most cytoplasmic membrane proteins do not contain a cleavable signal peptide and their N-terminal transmembrane segment serves as the membrane targeting signal. Most inner membrane proteins are targeted in a co-translational manner to the SecYEG translocase by the signal recognition particle (SRP) and its receptor (Herskovits *et al.*, 2000).

The bacterial SRP consists of a 48 kDa protein Ffh (Fifty-four homologue) and a 4.5S RNA (Keenan *et al.*, 2001; Luirink and Sinning, 2004). Together with its receptor FtsY, SRP delivers membrane and secretory proteins to the translocation channel in the plasma membrane. In *E. coli*, Ffh, FtsY and the 4.5S RNA are all essential for viability. Ffh consists of three domains: the N-terminal N domain (α-helical domain), the G domain (nucleotide binding domain) and the C-terminal M domain (methionine-rich, α-helical domain). SRP-binding substrates are bound by the methionine-rich M-domain of Ffh when they leave the ribosomal exit channel. There, a hydrophobic ‘finger loop’ of Ffh is proposed to interact with an α-helical substrate region, such as an uncleaved signal sequence (Luirink and Sinning, 2004). In *E. coli*, usually the first transmembrane region of multi-spanning membrane proteins interacts with SRP (Valent *et al.*, 1997; Beck *et al.*, 2000). The substrate specificity of SRP for membrane proteins may reflect the higher affinity of SRP for hydrophobic signal sequences. *In vitro* cross-linking studies have shown that the efficiency of cross-linking to Ffh is correlated with the hydrophobicity of the signal sequence (Valent *et al.*, 1997; 1998). Furthermore, *E. coli* presecretory proteins can be re-routed into the SRP pathway by increasing the hydrophobicity of their signal sequences (Lee and Bernstein, 2001).

In the SRP-mediated targeting pathway, SRP binds to the hydrophobic signal sequence of the nascent chain as it emerges from the translating ribosome. The resulting SRP–ribosome nascent chain complex is then targeted to FtsY at the membrane where an interaction involving GTP between SRP and its receptor catalyses the dissociation of the nascent chain from SRP. The nascent membrane proteins are then inserted into the SecYEG complex (for a review see Keenan *et al.*, 2001).

In the present study, we investigated in detail the initial interactions of the inner membrane protein KdpD with SRP. The membrane insertion of KdpD depends on SRP as we demonstrate with an Ffh- and an FtsY-depletion strain. We also show that a short sequence in the N-terminal cytoplasmic domain of the KdpD protein binds to SRP. A fusion protein of the green fluorescent protein (GFP) with KdpD is targeted to the membrane when the SRP-binding motif is present, but remains in the cytoplasm when this element is removed.

## Results

### Signal recognition particle is required for the membrane insertion of KdpD

**Fig. 2:**
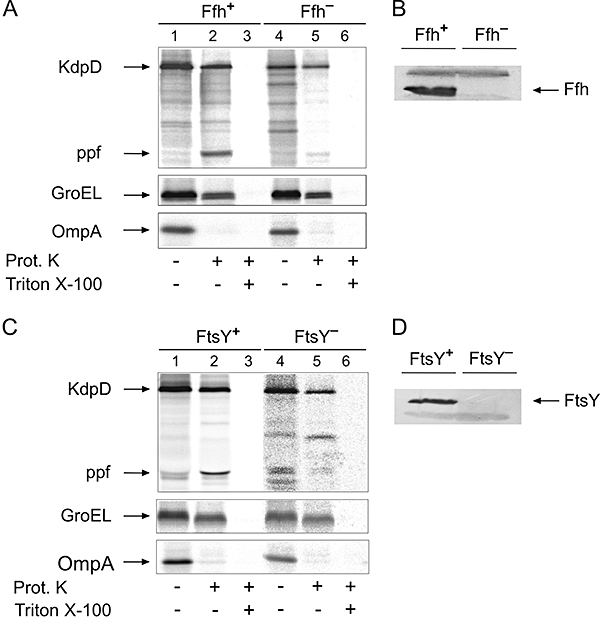
KdpD depends on SRP for targeting to the membrane. A. To test the requirement of SRP, the Ffh-depletion strain WAM121 was induced with arabinose or tightly repressed in the presence of glucose. *E. coli* strain WAM121 expressing KdpD (pSF51) was grown overnight in LB medium supplemented with arabinose. The cells were washed twice with LB medium, diluted 1:20 into fresh LB medium supplemented with arabinose (Ffh^+^) or glucose (Ffh^-^), and grown to an OD_600_ of 0.4. The cells were then transferred to M9 minimal medium and induced with 1 mM IPTG for 10 min. Cells were labelled with [^35^S]methionine for 1 min and chased with 500 μg ml^−1^ cold l-methionine for 2 min and subsequently converted to spheroplasts and incubated with (lanes 2 and 5) or without proteinase K (lanes 1 and 4) at a final concentration of 0.5 mg ml^−1^ on ice for 1 h. A lysis control was included by adding proteinase K and 2.5% Trition X-100 (lanes 3 and 6). All samples were precipitated with 20% TCA, immunoprecipitated with antisera against KdpD (upper panel), GroEL (middle panel) and OmpA (lower panel), and analysed by SDS-PAGE and visualized by phosphorimaging. B. Immunoblot analysis of Ffh levels in the WAM121 strain expressing KdpD under arabinose (Ffh^+^) and glucose (Ffh^-^) conditions. The samples were separated onto 12.5% SDS-PAGE and immunobloted with anti-Ffh antibody. C. To test the requirement of FtsY, the FtsY-depletion strain IY26 was induced with arabinose or tightly repressed in the presence of glucose. *E. coli* strain IY26 expressing KdpD was grown in LB medium with either arabinose (FtsY^+^) or glucose (FtsY^-^) for 4 h. The cells were then transferred to M9 minimal medium and induced with 1 mM IPTG for 10 min. Cells were pulse-labelled for 1 min and chased with cold l-methionine for 2 min and subsequently analysed as above. D. Immunoblot analysis of FtsY levels in the IY26 strain expressing KdpD under arabinose (FtsY^+^) and glucose (FtsY^-^) conditions. Immunoblot analysis was done using an FtsY antiserum. ppf, protease protected fragment.

The sensor kinase protein KdpD inserts into the membrane independently of the Sec translocase and YidC (Facey and Kuhn, 2003). To investigate the involvement of SRP in the biogenesis of KdpD, we transformed the Ffh-depletion strain WAM121 with the plasmid (pSF51) encoding the wild-type KdpD. In this strain, the *ffh* gene expression is under the control of the arabinose-inducible *araBAD* promoter (de Gier *et al.*, 1998). Ffh is depleted when the cells are grown in the presence of glucose and absence of arabinose. WAM121 cells were grown in the presence of glucose or arabinose, respectively. The cells were induced, labelled with [^35^S]methionine for 1 min, chased for 2 min, immediately converted to spheroplasts and treated with proteinase K. In a recent study, we observed that proteinase K did not cleave the protein in the first periplasmic loop P1, probably because of the short size of the loop (four amino acid residues). Cleavage in the second periplasmic loop P2 (10 amino acid residues) occurred only partially and led to a protease-protected fragment of 47 kDa which was recognized by the KdpD antibody (Facey and Kuhn, 2003). The results show that when Ffh was present, the translocation of the second periplasmic loop of KdpD was followed by the generation of the C-terminal 47 kDa proteolytic fragment (Fig. 2A, lane 2). In contrast, in Ffh-depleted cells, the formation of the C-terminal fragment was affected (compare lanes 2 and 5). Depletion of Ffh reduced the efficiency of the translocation of the second periplasmic loop of KdpD as indicated by the reduced appearance of the C-terminal fragment. This suggests that SRP is required for membrane insertion of KdpD. Following lysis of the cells with detergent, we confirmed that the smaller fragment was readily digested (lanes 3 and 6). As a control, the cytoplasmic protein GroEL and the outer membrane protein OmpA were analysed in parallel. The OmpA antibody used in this study recognizes the periplasmic domain of OmpA and the degradation of OmpA indicates that the protease is active in the periplasm. Depletion of the Ffh component of SRP largely inhibited KdpD insertion, without affecting the Sec-dependent export of OmpA. Ffh depletion was confirmed by analysing the Ffh content in a cell sample taken before induction with IPTG by immunoblotting using Ffh antiserum (Fig. 2B).

The membrane insertion of KdpD was further investigated in the FtsY-depletion strain IY26. Cells of strain IY26 in which *ftsY* expression is under control of an arabinose-inducible promoter were grown in the presence (FtsY^+^, Fig. 2C, lanes 1–3) or absence of arabinose (FtsY^-^, Fig. 2C, lanes 4–6) and then assayed for translocation. In accordance with the effects of depletion of Ffh, depletion of FtsY also inhibits membrane insertion of KdpD (compare lanes 2 and 5). Together, the *in vivo* results show that KdpD requires SRP for efficient membrane insertion. Furthermore, the processing of OmpA was unaltered when Ffh or FtsY were depleted, indicating that the Sec machinery was still functional. Depletion of FtsY was verified by analysing the FtsY level in a cell sample in parallel by immunoblotting with FtsY antiserum (Fig. 2D).

**Fig. 3:**
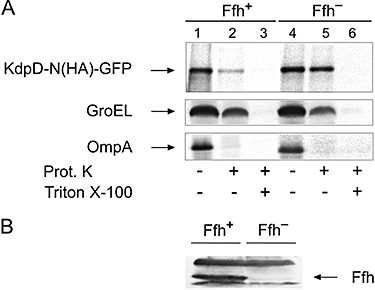
Protease accessibility of the epitope-tagged KdpD-N(HA)–GFP fusion protein in the Ffh-depletion strain WAM121. A. Cells expressing KdpD-N(HA)–GFP were grown in the presence of arabinose (Ffh^+^) or in the presence of glucose (Ffh^-^) and pulse-labelled for 1 min and chased for 2 min. Cells were then converted to spheroplasts and treated with or without proteinase K for 1 h, as described for Fig. 2A. The epitope-tagged KdpD-N(HA)–GFP fusion protein was immunoprecipitated with antiserum to HA (for the epitope in the first periplasmic loop) and then analysed as described for Fig. 2A. B. Immunoblot analysis of Ffh levels in the WAM121 strain expressing KdpD-N(HA)–GFP under arabinose (Ffh^+^) and glucose (Ffh^-^) conditions. Immunoblot analysis was done using antiserum to Ffh.

To test whether GFP fused to KdpD is also targeted by SRP, we analysed the translocation of a truncated KdpD fragment fused after amino acid residue 448 to GFP in the Ffh-depletion strain WAM121. The truncated fragment termed KdpD-N (i.e. coding the amino acids 1–448 of KdpD) has been described earlier (Facey and Kuhn, 2003). To analyse the translocation of this fragment, a short HA-epitope was introduced between helices 1 and 2 (Facey and Kuhn, 2003). It was previously shown that the first periplasmic loop of KdpD with an HA-epitope tag inserts into the inner membrane independent of YidC and the Sec components like wild-type KdpD (Facey and Kuhn, 2003). We investigated the effect of Ffh depletion on the translocation of KdpD-N fused to GFP in the strain WAM121. Here, the epitope-tagged KdpD-N(HA)–GFP fusion protein was immunoprecipitated with antiserum to HA. As described above, when Ffh was present (cells grown with arabinose), KdpD-N(HA)–GFP was readily inserted into the membrane and the exposed HA tag was digested with proteinase K (Fig. 3A, lanes 1 and 2). In Ffh-deficient cells (grown with glucose), the insertion of KdpD-N(HA)–GFP was inhibited. The protein was not accessible to the externally added proteinase K, indicating that it remains in the cytoplasm (Fig. 3A, compare lanes 4 and 5). These results are in agreement with the observations from the wild-type KdpD that SRP is required for membrane insertion. The fused GFP protein has no influence on the membrane targeting and insertion of KdpD. Interestingly, we show here that alone the N-terminal domain of KdpD requires SRP. Depletion of Ffh was confirmed by analysing the Ffh level in a cell sample in parallel by immunoblotting with Ffh antiserum (Fig. 3B).

### The KdpD–GFP fusion protein is localized at the bacterial membrane

**Fig. 4:**
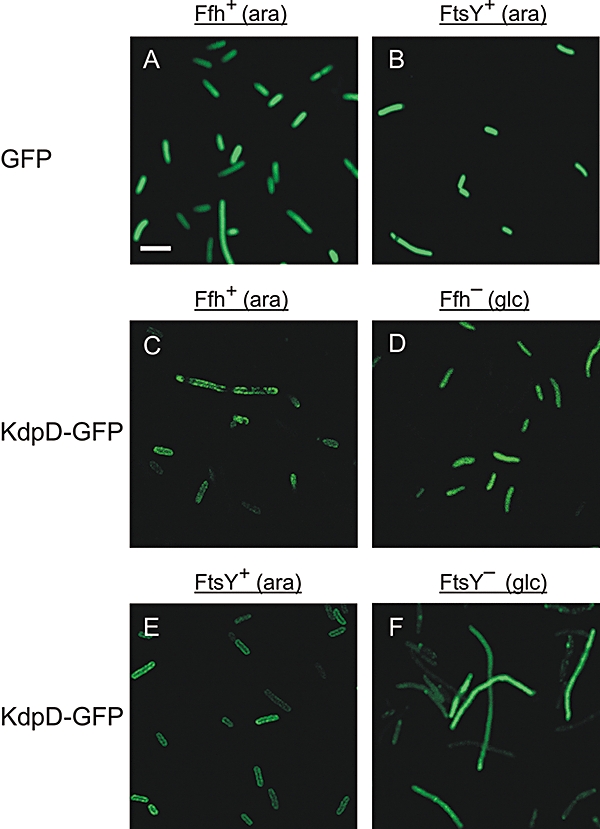
Localization of KdpD–GFP *in vivo* by fluorescence microscopy. Cells of strains WAM121 and IY26 bearing the GFP plasmid (A and B) or KdpD–GFP fusion plasmid (C-F) were grown in LB medium either in the presence of arabinose (Ffh^+^ FtsY^+^) or in the presence of glucose to deplete Ffh (D) or FtsY (F). Cells were induced with 1 mM IPTG for 1 h at 30°C. After induction, transcription was blocked by the addition of rifampicin (1 mg ml^−1^, final concentration) for 45 min. Cells were inspected under a fluorescence microscope as described in the *Experimental procedures* section. The bar represents 5 μm.

In order to visualize the localization of KdpD, we fused GFP to wild-type KdpD. To study the involvement of SRP in KdpD–GFP membrane targeting, we analysed the localization of KdpD–GFP in the Ffh-depletion strain WAM121 and FtsY-depletion strain IY26. When the cells expressing only GFP under arabinose conditions were examined by fluorescence microscopy, the fluorescence was uniformly distributed both in the WAM121 (Fig. 4A) and in the IY26 cells (Fig. 4B). Cells bearing the plasmid pJFBD/GFP coding for KdpD–GFP were grown under arabinose conditions (Ffh^+^ FtsY^+^) and examined by fluorescence microscopy, KdpD–GFP was found primarily located at the inner membrane (Fig 4C and E). However, when the cells were grown in the presence of glucose to deplete Ffh or FtsY (Fig 4D and F), the fusion protein was found uniformly located throughout the cytoplasm. These results are in agreement with the protease accessibility assay with wild-type KdpD that the SRP component Ffh and the SRP receptor FtsY are required for efficient targeting of KdpD to the membrane.

### Deletions in the N-terminal domain of KdpD affect the topology

**Fig. 5:**
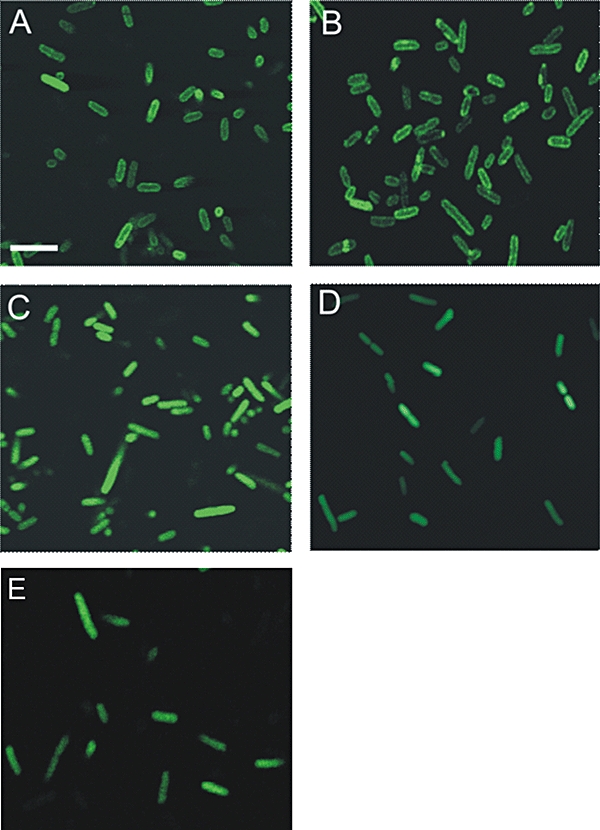
Deletion of the first 50 amino acids of KdpD inhibits its targeting to the membrane. *E. coli* MC1061 expressing KdpD–GFP (A), KdpD-N–GFP (B), KdpDΔ50–GFP (C), GFP (D) and KdpD-NΔ50–GFP (E) were examined by fluorescence microscopy. After induction, transcription was blocked by the addition of rifampicin (1 mg ml^−1^, final concentration) for 45 min. The bar represents 5 μm.

To gain insight into the signal sequence required for targeting of KdpD to the inner membrane with SRP, genetic variants were constructed with truncations in the N-terminal domain of KdpD–GFP. Because SRP acts at a very early stage in protein biosynthesis, one would expect the signal sequence to be at the beginning of a nascent chain because of co-translation of the protein binding to SRP. In most bacterial inner membrane proteins, the N-terminal transmembrane segment serves as the membrane targeting signal. However, the first transmembrane segment of KdpD is 400 amino acid residues from the N-terminus of KdpD. MC1061 cells were transformed with the plasmids coding for wild-type KdpD–GFP, KdpDΔ50–GFP, KdpD-N–GFP, KdpD-NΔ50–GFP and GFP (Fig. 5) and examined by fluorescence microscopy. The results show that when the first 50 N-terminal amino acid residues were deleted from KdpD–GFP, the fusion protein (KdpDΔ50–GFP) was distributed uniformly throughout the cytoplasm (Fig. 5C) like GFP alone (Fig. 5D). In contrast, more than 87% of total fluorescence of KdpD–GFP was located at the inner membrane (Fig. 5A). Similarly, KdpD-N–GFP composed of the cytoplasmic N-terminal region and the first two transmembrane regions of KdpD showed more than 99% of total fluorescence located at the membrane (Fig. 5B). In contrast, when the first 50 N-terminal amino acid residues were deleted from KdpD-N–GFP, the fusion protein (KdpD-NΔ50–GFP) was distributed uniformly throughout the cytoplasm (Fig. 5E).

**Fig. 6:**
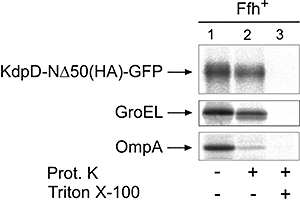
Deletion of the first 50 amino acid residues of KdpD impaired the insertion of KdpD into the membrane. *E. coli* WAM121 cells were transformed with the plasmid (pMS-NΔ50/GFP) containing the HA epitope-tagged KdpD-N protein (i.e. coding the amino acid residues 51–448 of KdpD) lacking the first 50 amino acid residues of KdpD fused to GFP. The cells were grown in the presence of arabinose as described in Fig. 2A. Cells were induced with 1 mM IPTG for 10 min, pulse-labelled with [^35^S]methionine for 1 min and chased with non-radioactive methionine for 2 min. The cells were then converted to spheroplasts and treated with or without proteinase K for 1 h. The epitope-tagged KdpD-N protein was immunoprecipitated with antisera to HA (for the epitope in the first periplasmic loop), GroEL and OmpA, and then analysed by SDS-PAGE and visualized by phosphorimaging.

The targeting of the N-terminal fragment of KdpD lacking the first 50 amino acid residues was investigated in the Ffh-depletion strain WAM121. To analyse the translocation of this fragment, a short HA-epitope was introduced between helices 1 and 2. Cells expressing the mutant protein (KdpD-NΔ50(HA)–GFP) were grown in medium supplemented with arabinose (Fig. 6). When Ffh was present, proteolysis of the N-terminal fragment of KdpD lacking the first 50 amino acid residues was inhibited (lane 2). Deletion of the first 50 amino acid residues of KdpD impaired the insertion of KdpD into the membrane. These results suggest that within the first 50 amino acid residues of KdpD, a sequence is present which targets KdpD to the inner membrane with SRP.

### A 48-residue-long peptide fused to GFP leads to its membrane localization but not its insertion

**Fig. 7:**
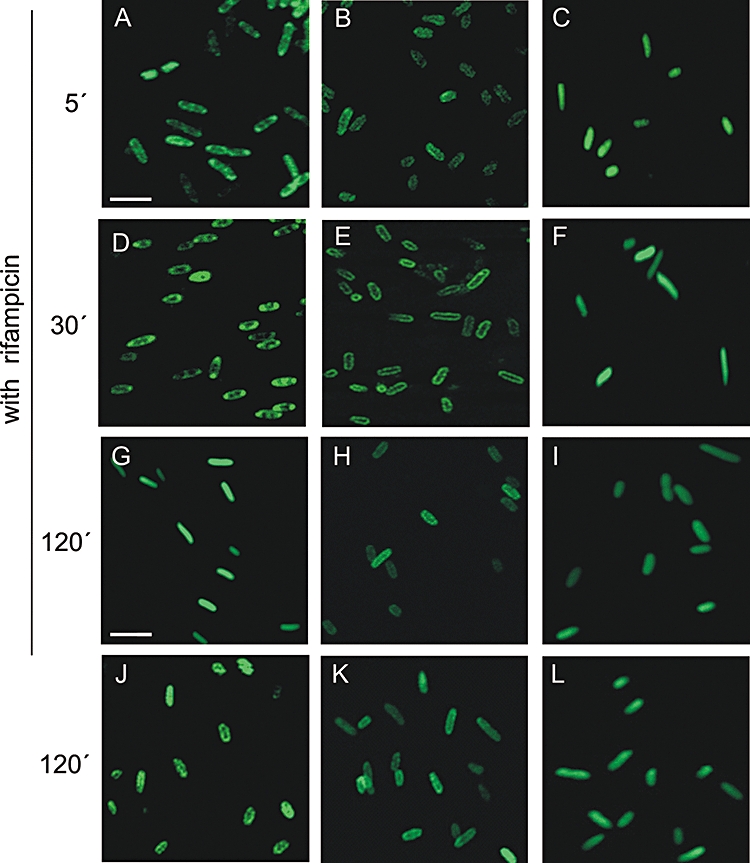
The first 48 amino acids of KdpD target GFP to the membrane. Localization of N48–GFP (A, D, G and J), KdpD–GFP (B, E, H and K), and N25–GFP (C, F, I and L) in wild-type *E. coli* strain MC1061. Synthesis of the GFP fusion proteins was induced either with IPTG for KdpD–GFP or arabinose for N25–GFP and N48–GFP. After induction for 1 h, rifampicin (1 mg ml^−1^, final concentration) was added and the localization of the fusion proteins was observed 5 min, 30 min and 2 h post rifampicin addition. The upper panel shows the fluorescence from the fusion proteins 5 min after treatment with rifampicin (A–C). The second panel shows the fluorescence from the fusion proteins 30 min after treatment with rifampicin (D–F). The third panel shows the fluorescence from the fusion proteins 2 h after addition of rifampicin (G–I). The lower panel shows the fluorescence from the fusion proteins without rifampicin after 2 h (J–L). The bars represent 5 μm.

To further characterize the SRP binding sequence for KdpD, additional *kdpD–gfp* gene fusions were constructed by fusing the first 25 and 48 N-terminal amino acid residues of KdpD to GFP to construct N25–GFP and N48–GFP, respectively. Synthesis of the GFP fusion proteins was induced either with IPTG for KdpD–GFP or arabinose for N25–GFP and N48–GFP for 1 h. At this point, the transcription inhibitor rifampicin was added to prevent further synthesis of the proteins. The localization of the fusion proteins was observed by fluorescence microscopy after 5 min (Fig. 7A–C), 30 min (Fig. 7D–F) and 2 h post rifampicin addition (Fig. 7G–I). Similar to the results seen with the full-length KdpD–GFP hybrid, transformants expressing N48–GFP (residues 1–48 of KdpD fused to GFP) showed a halo of fluorescence at the membrane in most of the cells at about 5 and 30 min after the addition of rifampicin (Fig. 7A and D). However, 2 h later, almost all the cells expressing N48–GFP showed the fusion protein uniformly distributed throughout the cytoplasm (Fig. 7G). These results imply that for N48–GFP, first the halo formation occurs in which N48–GFP is targeted to the inner membrane by the SRP pathway. Within the next 90 min, the protein is distributed back to the cytoplasm (Fig. 7G), most likely because no hydrophobic transmembrane region is present in the fusion protein, and therefore no insertion into the membrane can take place. We attempted to study the kinetics of membrane localization by following individual cells, but this was unsuccessful because the fluorescence was bleached after a few excitations. In contrast to N48–GFP, in cells expressing KdpD–GFP (Figs 7B, E and H) the membrane localization was kept at least for 2 h. Both proteins, N48–GFP and KdpD–GFP were not proteolytically digested during the 2 h as verified by Western blotting (data not shown). Cells expressing N25–GFP encoding the first 25 N-terminal amino acid residues of KdpD with GFP were found uniformly located throughout the cytoplasm at all expression times (Figs 7C, F and I).

A control is included to show what happens to the GFP fusion proteins in the absence of rifampicin addition without inhibiting the transcription and therefore protein synthesis (Fig. 7J–L). N48–GFP showed not only a halo of fluorescence at the membrane, but also the fusion protein uniformly distributed throughout the cytoplasm in the absence of rifampicin after 2 h (Fig. 7J). Cells expressing KdpD–GFP in the absence of rifampicin (Fig. 7K) showed the protein mainly located at the membrane like in the presence of rifampicin (Fig. 7H). When the N25–GFP fusion protein was examined by fluorescence microscopy without rifampicin (Fig. 7L), the fusion protein was found uniformly located throughout the cytoplasm like in the presence of rifampicin (Figs 7C, F and I).

**Fig. 8:**
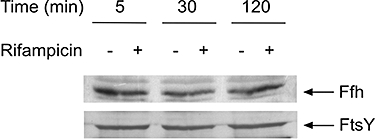
Western blot analysis of Ffh and FtsY levels in cells expressing the fusion proteins in the presence or absence of rifampicin. After induction for 1 h, cells were either treated with rifampicin (1 mg ml^−1^, final concentration) or without rifampicin for 5, 30 and 120 min as described in Fig. 7. Immunoblotting was done using antiserum against Ffh (upper panel) and FtsY (lower panel), respectively.

To exclude the possibility that the cytoplasmic localization of the GFP fusion proteins during the 2 h of rifampicin treatment is caused by a shortage of SRP components, the levels of Ffh and FtsY in the cells were verified by Western blot analysis. As shown in Fig. 8, Ffh and FtsY are still present at sufficient levels in the cells after rifampicin treatment. Therefore, the uniformly distribution of the GFP fusion proteins throughout the cytoplasm is not caused by a shortage of the SRP components, but influenced by the absence of transmembrane segments within the mutants which could insert and anchor the protein into the membrane. Taken together, these results show that with the use of rifampicin as a transcription inhibitor, we can follow the fate of the proteins after their synthesis.

**Fig. 9:**
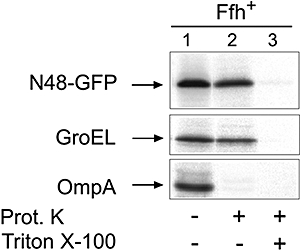
The first 48 amino acids of KdpD do not translocate GFP across the membrane. Cells of strain WAM121 (pSF165) were grown overnight in LB medium supplemented with arabinose. The overnight culture was diluted 1:20 into fresh LB medium supplemented with arabinose and grown to an OD_600_ of 0.4. The cells were then transferred to M9 minimal medium for 30 min. After induction with IPTG for 10 min, the cells were pulse-labelled with [^35^S]methionine for 1 min and chased with non-radioactive methionine for 2 min. Samples were prepared and processed as described for Fig. 2A. The N48–GFP protein was immunoprecipitated with antiserum against the N-terminus of KdpD.

To further test if N48–GFP encoding the first 48 N-terminal amino acid residues of KdpD with GFP is not translocated across the membrane, we introduced plasmid pSF165 coding for N48–GFP into the *E. coli* strain WAM121. When WAM121 (pSF165) was grown in LB with 0.2% (w/v) arabinose, N48–GFP accumulated in the cytoplasm, showing that the first 48 N-terminal amino acid residues of KdpD are not capable to secrete the fusion protein across the membrane (Fig. 9). As expected, the fusion protein is not integrated into the membrane because no hydrophobic transmembrane segment is present. Our results indicate that the region of the first 48 amino acid residues of KdpD is required for SRP-dependent targeting, whereas the transmembrane regions of KdpD are required for insertion into the membrane.

**Fig. 10:**
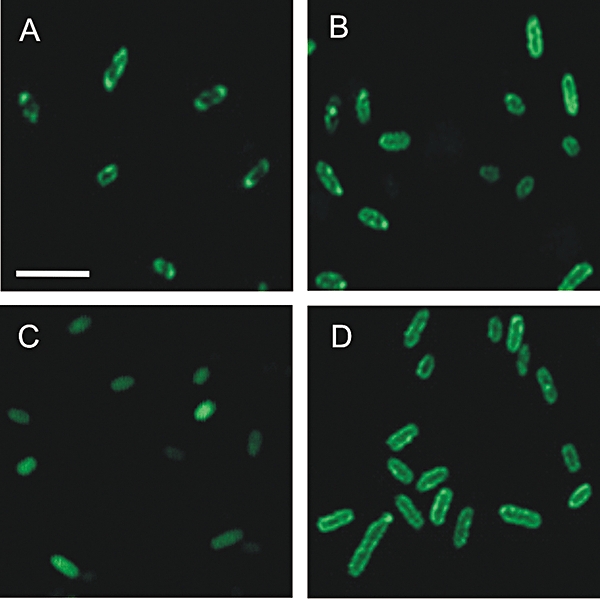
The amino acids 22–48 of KdpD target GFP to the membrane. Localization of N22–48/GFP in *E. coli* MC1061 5 min (A), 30 min (B) and 2 h (C) after treatment with rifampicin as described in Fig. 7. D shows the fluorescence from N22–48/GFP without rifampicin after 2 h. The bar represents 5 μm.

We then generated a smaller construct containing the amino acid residues 22–48 of KdpD fused to GFP. This plasmid coding for N22–48/GFP was transformed in *E. coli* MC1061. Synthesis of the N22–48/GFP fusion protein was induced with arabinose and cells were grown for 1 h. At this point, the transcription inhibitor rifampicin was added to prevent further synthesis of the GFP fusion proteins and the localization of N22–48/GFP was observed by fluorescence microscopy after either 5 min (Fig. 10A), 30 min (Fig. 10B) or 2 h (Fig. 10C). When the N22–48/GFP fusion protein was examined by fluorescence microscopy after 5 and 30 min, the fusion protein was primarily located at the inner membrane (Fig. 10A and B). It appears that like N48–GFP, newly synthesized N22–48/GFP is first targeted to the membrane and then transferred back to the cytoplasm (Fig. 10C). The absence of a transmembrane region inhibits insertion of the fusion protein into the membrane. This result is consistent with the notion that SRP is required for membrane targeting and subsequently the transmembrane regions of KdpD for insertion into the membrane. N22–48/GFP was not proteolytically digested during the 2 h as verified by Western blotting (data not shown). Without rifampicin addition, cells expressing N22–48/GFP showed the fusion protein not only located at the membrane but also uniformly distributed throughout the cytoplasm (Fig. 10D), like for N48–GFP (Fig. 7J).

### The 48-residue peptide of KdpD binds to SRP

**Fig. 11:**
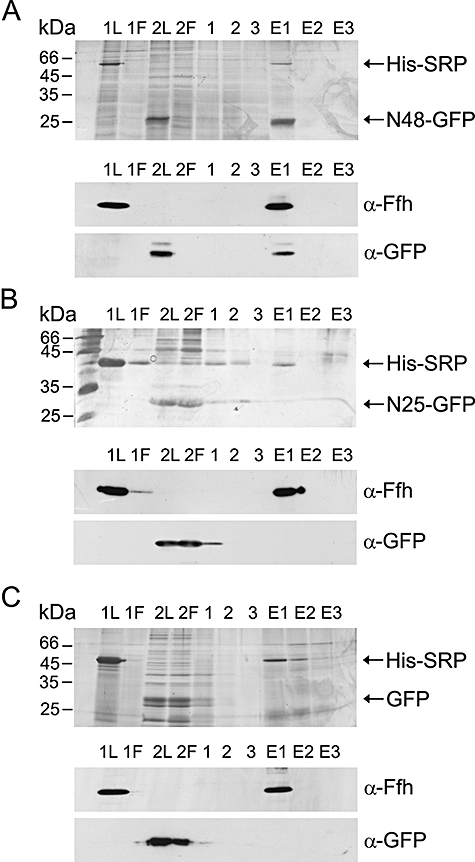
Interaction of the amino acid residues 1–48 of KdpD with His-SRP. Pull-down assays of N48–GFP (A), N25–GFP (B) and GFP (C) with His-SRP. About 5 μg of His-SRP protein (Ffh + 4.5S RNA) was attached to a Ni Sepharose column (lane 1L) which had been pre-equilibrated with 1.5 ml of equilibration buffer as indicated in *Experimental procedures*. Unbound protein was collected by centrifugation (lane 1F). Purified GFP and GFP fusion proteins (∼5 μg) were incubated with immobilized His-SRP for 1 h at 4°C (lane 2L). Again unbound protein was collected by centrifugation (lane 2F). After three washes (lanes 1–3), bound proteins were eluted with 500 mM imidazole (lanes E1–3) and separated by SDS-PAGE. Gels were silver-stained (upper panels). Bound proteins were identified by Western blotting with anti-Ffh (middle panels) and anti-GFP (lower panels). L, load; F, flow-through; E, elution.

To test whether the N-terminal signal element is capable of directly binding to SRP *in vitro*, we investigated the interaction between N48–GFP (residues 1–48 of KdpD fused to GFP) and N25–GFP (residues 1–25 of KdpD fused to GFP) with the His-SRP protein by pull-down assays. To carry out these experiments, the GFP fusion proteins were expressed in *E. coli* MC1061 and purified by immobilized metal affinity chromatography (Ni-IMAC). GFP contains 10 histidine residues which interact with Ni(II) ions (Li *et al.*, 2001). To avoid GFP also binding to the Ni Sepharose column for the pull-down assays, the GFP fusion proteins were added to the column in equilibration buffer containing 25 mM imidazole. In the assay, the His-SRP protein was attached to the Ni Sepharose column (Fig. 11, lane 1L) and then incubated with one of the purified GFP fusion proteins (Fig. 11, lane 2L), and after extensive washing (Fig. 11, lanes 1–3), the bound proteins were eluted (Fig. 11, lanes E1–3) and analysed by SDS-PAGE and Western analysis. As shown in Fig. 11A, we found that the N48–GFP protein co-elutes from the resin with the His-tagged SRP protein (lane E1), demonstrating that the first 48 residues of KdpD physically interact with SRP. In contrast, the N25–GFP protein was unable to bind and co-elute from the resin with the His-tagged SRP protein (Fig. 11B, lane E1). As a control and evidence of the specificity of this interaction, GFP did not bind to the His-SRP protein (Fig. 11C).

As negative controls, GFP, N25–GFP and N48–GFP were added to the column without His-SRP. None of the proteins were able to bind specifically to the Ni Sepharose column under the assay conditions we used (data not shown).

These results show that the amphiphilic region including residues 22–48 of KdpD interacts directly with SRP.

## Discussion

The membrane targeting of most newly synthesized integral proteins is mediated by SRP. As in *E. coli* nearly all membrane proteins have an N-terminal uncleavable signal peptide, the region that interacts with SRP is most likely the first transmembrane region. This ensures that early after the onset of translation, the nascent protein interacts with SRP and targets the protein co-translationally to the membrane (for a review see Luirink and Sinning, 2004). In the sensor protein KdpD, the first transmembrane region starts 401 residues after the initiating methionine preventing an early membrane targeting of the protein. Therefore, an additional signal element might be present at the N-terminus to allow co-translational membrane targeting. Our results show that the residues 22–48 of KdpD contain a signal element that is capable to bind to SRP and to target the GFP protein to the membrane.

GFP and its mutants have become an invaluable marker for monitoring protein localization and gene expression *in vivo* (for a review see Margolin, 2000). Using GFP fused to KdpD, we examined where the targeting information of KdpD to the inner membrane is localized. When the 400-residue-long hydrophilic domain with helices 1 and 2 (KdpD-N) was fused to GFP, we found that the fusion protein was found at the membrane (Fig. 5B). This suggested that the information for membrane targeting might lie within the first 448 residues of KdpD. Moreover, a deletion of the first 50 amino acid residues of KdpD impaired the insertion of KdpD (Fig. 5C) and KdpD-N (Fig. 5E) into the membrane. We then fused the first 48 amino acid residues of KdpD to GFP and found that this protein was readily targeted to the membrane (Fig. 7A and D). Taken together, these results show that an N-terminal signal element is required for the targeting of KdpD to the inner membrane. Although the most direct explanation of these data is that SRP targets KdpD to the membrane, it is possible that a protein targeted to the membrane by SRP is required for KdpD targeting.

**Fig. 12:**
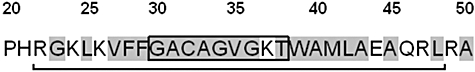
The amino acid sequence of the N-terminal signal element that is required for targeting of KdpD to the inner membrane. The minimal peptide of 22–48 is underlined. Hydrophobic amino acids are shaded dark. Marked with a box resembles the sequence of a Walker A motif.

The results provided here clearly show that SRP (Ffh) and the SRP receptor (FtsY) are involved in the membrane targeting of KdpD. *E. coli* strains that can be manipulated to deplete SRP or its receptor show that KdpD insertion is impaired in the absence of Ffh (Fig. 2A) or FtsY (Fig. 2C). Also, the KdpD deletion mutant lacking the first 50 amino acid residues was incapable of inserting into the membrane as judged by the protease mapping technique (Fig. 6). These observations suggested that the N-terminal signal element of KdpD directly binds to SRP. Indeed, His-SRP was binding to the purified fusion protein N48–GFP that contained the signal element (Fig. 11A). The minimal peptide that supported the binding was N22–48 fused to GFP. This peptide (Fig. 12) contains five positively charged residues, in which three are closely spaced (aa 22–26) and a stretch of 10 hydrophobic residues (aa 27–36) followed by another six hydrophobic residues (aa 38–43). In general, a highly hydrophobic signal anchor region is the binding element for SRP-mediated co-translational membrane insertion of integral membrane proteins (de Gier *et al.*, 1998; Peterson *et al.*, 2003). In addition, basic amino acids promote binding of the signal peptide with SRP through electrostatic interactions (Peterson *et al.*, 2003). The crystal structure of a complex between the *E. coli* Ffh M domain and a fragment of the 4.5S RNA has revealed an unusual RNA–protein interface that is thought to constitute the signal peptide binding groove (Batey *et al.*, 2000). It has been suggested that this binding site composed of both protein and RNA would accommodate a signal peptide through a combination of hydrophobic interactions and electrostatic contacts. Therefore, the three closely spaced basic residues (Arg and Lys) in the peptide might enhance the SRP binding and compensate the lower hydrophobicity of the KdpD signal element. It would be interesting to exchange the lysyl and arginyl residues in the peptide into neutral amino acid residues to see if the peptide still interacts with SRP. Within this peptide, a Walker A motif was found from residues 30–38 (Jung and Altendorf, 1998). This motif is very similar to the classical ATP binding site of many ATP-requiring enzymes (Walker *et al.*, 1982). It is conceivable that shortly after its exit from the ribosome, the SRP binds to the motif and delivers the protein to the membrane where it is released. The KdpD protein is then folded and the Walker A motif is subsequently used for an ATP-binding site. The N-terminal KdpD peptide is the first case that a non-transmembrane sequence provides binding to SRP and is capable to target the hydrophilic GFP to the membrane surface. As the SRP-signal peptide from KdpD is soluble, it provides an attractive tool for future SRP binding studies. The Ffh and FtsY dependence of KdpD is a further indication that the N-terminal peptide is functional in membrane targeting.

Analysis of the first 48 amino acid residues of KdpD fused to GFP indicates accumulation mainly at the inner membrane but not insertion into the membrane (Fig 7A and D, Fig 9). We assume that after contacting FtsY, GTP is rapidly hydrolysed and the GFP fusion protein is released from SRP. With no transmembrane regions for anchoring into the membrane, the protein slowly distributes throughout the cytoplasm after some time (Fig. 7G). It appears that the cells show a punctuate fluorescence staining which might reflect the formation of protein clusters at the cell periphery (Figs 4C, Fig 10A and B). The N-terminal KdpD peptide is required for SRP-dependent targeting, whereas the transmembrane regions of KdpD are required for insertion into the membrane. The data here are consistent with a co-translational targeting mechanism of KdpD *in vivo*. Usually, SRP binds to signal sequences of nascent polypeptides as they emerge from the ribosome. It has been proposed that the nature of a nascent polypeptide is already sensed in the ribosome, which may influence the binding of SRP near the nascent chain exit site (Gu *et al.*, 2003; Ullers *et al.*, 2003). Previous studies have shown that the *E. coli* inner membrane protein, Leader peptidase (Lep), is targeted co-translationally by the SRP to the Sec-YidC insertion site in the membrane (de Gier *et al.*, 1996; 1998; Samuelson *et al.*, 2000). Photo cross-linking studies have shown that nascent Lep with a length of 40 amino acids and longer could be immunoprecipitated using anti-Ffh (Houben *et al.*, 2002; 2005). Data suggest that targeting of Lep with SRP to the Sec-YidC insertion site starts at an early stage during biosynthesis of Lep when the nascent chain is 40 amino acids long. This would be in agreement with our results that SRP binds remarkably early during biogenesis of KdpD.

As most of the integral membrane proteins use the SecYEG translocase pathway, it was first assumed that the targeting pathways mediated by SRP converge at the translocase (Valent *et al.*, 1998). Indeed, experimental evidence exists that show an interaction between SRP and SecYEG (Weiche *et al.*, 2008). Cross-linking studies have demonstrated for a number of proteins a consecutive binding to SRP and Sec (Valent *et al.*, 1998; Beck *et al.*, 2000). More recently, it was shown that the Sec-independent protein MscL is targeted by SRP, but uses YidC for membrane insertion (Facey *et al.*, 2007). Another example is KdpD, which translocates its two extremely short periplasmic loops in the absence of the Sec translocase and YidC (Facey and Kuhn, 2003). This underlines that targeting and insertion/translocation are functioning as separate modules and cooperate depending on the actual substrate protein.

## Experimental procedures

### Bacterial strains and culture conditions

Microscopy experiments were performed with *E. coli* MC1061 [*hsdR mcrB araD139* (*araABC-leu*)*7697 lacX74 galU galK rspL thi*] (Meissner *et al.*, 1987).

For Ffh depletion, cells of *E. coli* strain WAM121 (de Gier *et al.*, 1998) were grown overnight in LB medium containing 0.2% (w/v) arabinose, washed in medium lacking arabinose and back-diluted 1:20 in LB medium containing 0.4% (w/v) glucose. When cultures reached an OD_600_ of 0.4, the cells were transferred to M9 minimal medium for 30 min prior to pulse-chase labelling.

The FtsY-depletion strain IY26 (BW25113-Kan-AraCP-ftsY) was obtained from I. Yosef and E. Bibi. FtsY is under the control of the *araBAD* promoter and operator. The FtsY-depletion strain IY26 was grown in LB medium supplemented with 0.2% (w/v) arabinose. For FtsY depletion, overnight grown cultures were first washed twice with LB medium and then back-diluted 1:40 and were grown to an OD_600_ of 0.4 (∼4 h) in LB medium with 0.2% (w/v) glucose. Medium was switched to M9 minimal medium containing glucose and the cells were grown for an additional 30 min before labelling.

Media preparation and bacterial manipulations were performed according to standard methods (Maniatis *et al.*, 1982). Where appropriate, ampicillin (100 μg ml^−1^, final concentration), kanamycin (30 μg ml^−1^, final concentration) or chloramphenicol (25 μg ml^−1^, final concentration) were added to the medium.

### Construction of GFP fusion proteins

The plasmids used in this study are described in Table 1. K. Jung and K. Altendorf kindly provided the plasmid pBD carrying the *kdpD* gene in pBAD18 (Jung and Altendorf, 1998). A *kdpD–gfp* fusion gene where the last codon of *kdpD* was fused to codon 1 of *gfp* mut3.1 (Cormack *et al.*, 1996) was constructed as follows. Plasmid pJDT1 (kindly provided by S. Thompson and C. Robinson) is a pBAD24 derivative carrying a *torA–gfp* fusion (Thomas *et al.*, 2001). A unique SphI site was introduced by site-directed mutagenesis at the end of the *torA* gene. A ∼1.4 kb DNA fragment containing the *kdpD-C* gene (i.e. encoding residues of 444–894 of KdpD) lacking the translation stop codon was obtained by PCR and cloned as an MluI-SphI fragment into the MluI-SphI-digested plasmid pJDT1/SphI to construct p24C/GFP (the *torA* gene was replaced with the *kdpD-C* gene). Plasmid p24C/GFP containing the *kdpD-C–gfp* chimeric gene was cleaved with StuI/HindIII and inserted in StuI-HindIII-digested pBD to generate pBD/GFP encoding KdpD and GFP.

**Table 1:** Constructs and vectors used in this study.

Plasmids	Name	Characteristics	Reference or source
pBAD33		Cm^R^, *araC*, P_BAD_, pACYC *ori*, expression vector	Guzman *et al.* (1995)
pMS119EH		Ap^R^, P_tac_, expression vector	Balzer *et al.* (1992)
pJF119EH		Ap^R^, P_tac_, expression vector	Fürste *et al.* (1986)
pT7-7		Ap^R^, T7 promoter, expression vector	Tabor and Richardson (1985)
pGFPmut3.1	GFP	Ap^R^, *gfp* mut3.1	Clontech
pJDT1	TorA–GFP	Ap^R^, pBAD24, *torA–gfp*	Thomas *et al.* (2001)
pBD	KdpD	Ap^R^, pBAD18, *kdpD*	Jung and Altendorf (1998)
pSF18	KdpD-N(HA)	Ap^R^, pT7-7, *kdpD*_Δ449−894_	Facey and Kuhn (2003)
pSF51	KdpD	Ap^R^, pMS119EH, *kdpD*	This study
pBD/GFP	KdpD–GFP	Ap^R^, pBAD18, *kdpD–gfp*	This study
pJFBD/GFP	KdpD–GFP	Ap^R^, pJF119EH, *kdpD–gfp*	This study
pBDΔ50/GFP	KdpDΔ50–GFP	Ap^R^, pBAD18, *kdpD*_Δ2−50_*–gfp*	This study
p33/GFP	GFP	Cm^R^, pBAD33, *gfp*	This study
p33N/GFP	KdpD-N–GFP	Cm^R^, pBAD33, *kdpD*_Δ449−894_*–gfp*	This study
p24C	KdpD-C	Ap^R^, pBAD24, *kdpD*_Δ1−443_	This study
p24C/GFP	KdpD-C–GFP	Ap^R^, pBAD24, *kdpD*_Δ1−443_*–gfp*	This study
pSH-N/GFP	KdpD-N(HA)–GFP	Cm^R^, pBAD33, *kdpD*_Δ449−894_*–gfp*	This study
pMS-N/GFP	KdpD-N(HA)–GFP	Ap^R^, pMS119EH, *kdpD*_Δ449−894_*–gfp*	This study
pT7-NΔ50(HA)	KdpD-N Δ50(HA)–GFP	Ap^R^, pT7-7, *kdpD*_Δ2−50; Δ449−894_	This study
pSH-NΔ50/GFP	KdpD-N Δ50(HA)–GFP	Cm^R^, pBAD33, *kdpD*_Δ2−50; Δ449−894_*–gfp*	This study
pMS-NΔ50/GFP	KdpD-N Δ50(HA)–GFP	Ap^R^, pMS119EH, *kdpD*_Δ2−50; Δ449−894_*–gfp*	This study
pBAD-N25/GFP	N25–GFP	Cm^R^, pBAD33, *kdpD*_Δ26−894_*–gfp*	This study
pBAD-N48/GFP	N48–GFP	Cm^R^, pBAD33, *kdpD*_Δ49−894_*–gfp*	This study
pBAD-N22–48/GFP	N22–48/GFP	Cm^R^, pBAD33, kdpDΔ_2−21; Δ49−894_*–gfp*	This study
pSF163	N25–GFP	Ap^R^, pMS119EH, *kdpD*_Δ26−894_*–gfp*	This study
pSF165	N48–GFP	Ap^R^, pMS119EH, *kdpD*_Δ49-894_*–gfp*	This study

To generate the plasmid pJFBD/GFP, the coding region of KdpD–GFP was excised by SmaI-HindIII from pBD/GFP and cloned into the corresponding sites of the plasmid pJF119EH. Plasmid pBDΔ50/GFP was constructed in several steps. PCR amplification with the primer set NcoI-50-KdpD (5′-GCATACCATGGCGCAAGGGCTGGATATTGTGGTTG-3′), and StuI-KdpD (5′-GCAGGCAGGCCTTTATCAAAACTCCACTGCG-3′) and with pBD as the template produced a ∼1.6 kb NcoI-StuI fragment. This fragment was subsequently inserted into the NcoI-StuI site of p24C (i.e. encoding residues of 444–894 of KdpD). The resulting plasmid was digested with MluI and SphI and ligated to MluI-SphI-digested pBD/GFP to create pBDΔ50/GFP.

Construction of pSH-N/GFP was done as follows: *gfp* mut3.1 was amplified by PCR from pGFPmut3.1 (Clontech) with the primers GFP-PstI (5′-CCGTCTGCAGATGCGTAAAGGAGAAGAACTTTTC-3′) and GFP-HindIII (5′-CCGGCAAGCTTATTTGTATAGTTCATCCATGC-3′). From this PCR product, a 0.72 kb PstI-HindIII fragment carrying the *gfp* mut3.1 gene was isolated and subsequently ligated to PstI-HindIII-digested pBAD33 to create p33/GFP. The plasmid pSF18 encoding KdpD-N (i.e. encoding residues 1–448 of KdpD with the HA-epitope) has been described (Facey and Kuhn, 2003). In this plasmid, the stop codon (TAG) was changed to GGG (glycine) by site-directed mutagenesis. To construct pSH-N/GFP, the sequence encoding KdpD-N lacking its stop codon was cleaved with XbaI-PstI and cloned into XbaI-PstI-digested p33/GFP. To generate the plasmid pMS-N/GFP, the coding region of KdpD-N(HA)–GFP was excised by XbaI-HindIII from pSH-N/GFP and inserted into the corresponding sites of pMS119EH.

Primers KdpD-N-50 (5′-GAATGAATTACATATGGCGCAAGGGCTGGATATTG-3′) and KdpD-N-PstI (5′-AAATCTG CAGCTATGAAGGCCAGCGTCCATAAAATA-3′) were used to amplify a ∼1.25 kb fragment from the plasmid pSF18 encoding KdpD-N (Facey and Kuhn, 2003). This fragment was digested with NdeI and PstI and cloned into the corresponding sites of pT7-7, to generate the plasmid pT7-NΔ50(HA). By means of site-directed mutagenesis, the stop codon (TAG) was changed to GGG (glycine). The resulting plasmid pT7-NΔ50(HA)/Gly was cleaved with XbaI-PstI and inserted into XbaI-PstI-digested p33N/GFP to create pSH-NΔ50/GFP encoding KdpD-NΔ50(HA)–GFP. To construct pMS-NΔ50/GFP, plasmid pSH-NΔ50/GFP was cleaved with XbaI-HindIII and cloned into the corresponding sites of pMS119EH. Plasmid pBD containing KdpD was cleaved with SmaI-HindIII and the fragment coding for the *kdpD* gene was inserted into SmaI-HindIII-digested pMS119EH to generate pSF51. Plasmids pBAD-N25/GFP and pBAD-N48/GFP were constructed from p33N/GFP encoding KdpD-N and GFP in pBAD33. A second PstI site was introduced by site-directed mutagenesis into p33N/GFP after the first 25 and 48 amino acid residues of KdpD, respectively. The deletion derivatives were made by digesting p33N/GFP/PstI with PstI and re-ligating the appropriate gel-purified fragments creating pBAD-N25/GFP and pBAD-N48/GFP. To generate the plasmids, pSF163 and pSF165, the coding region of N25–GFP and N48–GFP was excised by XbaI/HindIII from pBAD-N25/GFP and pBAD-N48/GFP and inserted into the corresponding sites of pMS119EH, respectively. N22–48/GFP, containing amino acid residues 22–48 of KdpD fused to GFP, was constructed as follows: amino acids 2–21 were deleted by site-directed mutagenesis in pBAD-N48/GFP. The coding regions of all constructs were verified by sequence analysis.

### Fluorescence microscopy

Strains were grown overnight at 37°C, diluted 1:20 in fresh LB medium, and grown to an OD_600_ of 0.4. IPTG was then added to a final concentration of 1 mM, and 0.2% (w/v) arabinose was added in strains carrying *kdpD* genes under the control of the *araBAD* promoter and operator. The cells were incubated for 30 min to 1 h at 30°C under continuous shaking. Rifampicin (1 mg ml^−1^, final concentration) was added, and the cultures were incubated at 30°C for the times indicated under *Results*. It was verified that under these conditions, the expression of the KdpD–GFP fusion proteins was inhibited. Cells were either collected by centrifugation, washed twice with LB medium and re-suspended in 2 mM EDTA, 50 mM Tris-HCl, pH 8.0 or treated with trichloroacetic acid (TCA) (10%, final concentration) and analysed by Western blotting. A cell suspension (3 μl) was applied to a polylysine-coated cover glass (Sigma-Aldrich) and the cells were examined immediately by Zeiss LSM 510 Meta, confocal fluorescence microscope. Emission was detected with a filter specific for GFP.

### Pulse-chase and immunoprecipitation analyses

For all experiments, cells were grown to midlogarithmic phase. Cells harbouring the plasmid-encoded proteins were induced for 10 min with IPTG (1 mM, final concentration). Cells were then labelled with 10 μCi ml^−1^ of [^35^S]methionine for 1 min and chased with excess l-methionine for 2 min. For spheroplasting, cells were centrifuged and re-suspended in 500 μl of ice-cold spheroplast buffer [40% (w/v) sucrose, 33 mM Tris-HCl, pH 8.0]. Lysozyme (5 μg ml^−1^, final concentration) and EDTA (1 mM, final concentration) were added for 15 min. Aliquots of the spheroplast suspension were incubated on ice for 1 h either in the presence or absence of proteinase K (0.5 mg ml^−1^, final concentration). A lysis control was included by adding 2.5% Triton X-100 and proteinase K for 1 h. After addition of phenylmethylsulfonyl fluoride (0.33 mg ml^−1^, final concentration), samples were precipitated with TCA (20%, final concentration), re-suspended in 10 mM Tris/2% SDS, pH 8.0 and immunoprecipitated with antibodies against HA, KdpD, OmpA (a periplasmic control), or GroEL (a cytoplasmic control). Samples were analysed by SDS-PAGE and phosphorimaging.

### Expression and purification of GFP fusion proteins

*Escherichia coli* strain MC1061 harbouring the plasmid pGFPmut3.1 (Clontech) or pBAD-N25–GFP or pBAD-N48–GFP was grown at 30°C in LB medium (1 l) with 100 μg ml^−1^ ampicillin or 25 μg ml^−1^ chloramphenicol, respectively. When an OD_600_ of 0.5 was reached, the cells were induced either with IPTG (1.0 mM, final concentration) or arabinose (0.2%, final concentration), respectively, and grown for an additional 10 h at 30°C. Cells were harvested by centrifugation and pellets were re-suspended in 0.05 M sodium phosphate buffer, pH 7.5, 0.25 M NaCl and lysed with a French press. The cell debris was removed by centrifugation. The filtered extract was applied to IMAC column containing Chelating Sepharose 6B resin (Pharmacia) charged with Ni(II) ions, which had been pre-equilibrated with 0.05 M sodium phosphate buffer, pH 7.5, 2 M NaCl as described by Li *et al.* (2001). Unbound protein was removed from the column by washing with 10 column volumes of equilibration buffer. Protein was eluted from the column with a falling pH (7.5–4.0) in a 0.05 M sodium phosphate buffer and 2 M NaCl. The purification process was monitored by SDS-PAGE analysis.

### Pull-down assays

The purified His-SRP protein (Ffh + 4.5S RNA) was kindly provided by I. Sinning. For pull-down assays, about 5 μg of His-SRP protein was attached to a column containing 300 μl of Ni Sepharose High Performance (Amersham Biosciences) which had been pre-equilibrated with 1.5 ml of equilibration buffer (15 mM Na_2_HPO_4_, 150 mM NaCl, 10% glycerol, 25 mM imidazole, pH 7.6) for 1 h at 4°C. Unbound protein was collected by centrifugation. GFP-fused proteins (∼5 μg) were added to the immobilized His-SRP protein and were incubated for 1 h at 4°C. After extensive washing with 1.8 ml of equilibration buffer containing 25 mM imidazole, bound proteins were eluted with 500 mM imidazole. The expected size of the co-eluted proteins were verified by SDS-PAGE (15%) and Western blot analysis.
